# Supplementary material for: Variations in Yield, Essential Oil, and Salicylates of Filipendula ulmaria Inflorescences at Different Blooming Stages
Source: Plants (Basel). 2023 Jan 8;12(2):300. doi: 10.3390/plants12020300 (PMC9862637; doi:10.3390/plants12020300)
Supplement: Supplementary file 1 [file plants-12-00300-s001.zip › plants-2110877-supplementary.pdf]

Supplementary Table S1. Essential oil composition of *Filipendula ulmaria* inflorescences collected from different habitats in early and late blooming stages.

Compound name	$RI^{Lit.}/RI^{Calc.}$	Habitat									
		Biržai		Pabiržė		Kernavė		Vilnius		Mikniškės	
		early stage	late stage	early stage	late stage	early stage	late stage	early stage	late stage	early stage	late stage
		Content (%)									
(2E)-Heptenal	954/954	nd.	nd.	nd.	0.1	nd.	tr.	nd.	tr.	tr.	0.1
Benzaldehyde	960/960	0.2	nd.	nd.	tr.	nd.	0.1	0.1	0.2	0.2	0.2
6-methyl-Hept-5-en-2-one	985/986	nd.	nd.	nd.	0.1	nd.	tr.	nd.	nd.	nd.	tr.
2-Penthyl furan	988/989	tr.	tr.	0.1	0.2	nd.	0.1	tr.	0.1	tr.	0.1
Octanal	998/998	tr.	nd.	tr.	0.1	nd.	0.1	tr.	0.1	0.1	0.1
Salicylaldehyde	1039/1040	79.7	94.8	68.9	39.6	71.3	76.8	73.6	57.8	67.9	59.3
Octanol	1068/1069	nd.	nd.	tr.	0.3	nd.	0.1	nd.	0.2	nd.	0.2
cis-Linalool oxide	1072/1073	tr.	nd.	nd.	nd.	nd.	tr.	tr.	nd.	0.1	nd.
Linalool	1096/1096	0.7	0.5	0.9	1.3	0.6	nd.	0.7	1.7	0.9	1.0
Nonanal	1100/1100	1.5	2.2	2.0	5.0	1.4	3.7	1.5	3.1	2.3	3.2
Lilac aldehyde A	1152/1153	0.3	nd.	0.2	0.1	nd.	0.1	0.4	0.2	0.5	0.3
Lilac aldehyde B	1154/1154	0.4	tr.	0.3	nd.	0.2	0.1	0.7	0.3	0.8	0.4

Unknown	-/1157	nd.	nd.	nd.	nd.	nd.	0.1	nd.	0.1	nd.	0.1
Lilac aldehyde C	1159/1160	nd.	nd.	nd.	0.1	0.3	nd.	nd.	nd.	nd.	nd.
(2E)-Nonen-1-al	1161/1161	nd.	nd.	tr.	0.2	nd.	0.1	tr.	0.1	tr.	0.1
Lilac aldehyde D	1161/1161	0.2	nd.	0.1	0.1	nd.	0.1	0.3	0.1	0.3	0.2
Nonanol	1172/1172	nd.	nd.	nd.	0.1	nd.	0.1	nd.	nd.	nd.	0.1
α-Terpineol	1188/1189	nd.	0.2	nd.	nd.	0.1	nd.	nd.	nd.	nd.	nd.
Methyl salicylate	1190/1190	14	tr.	20.1	24.5	21.5	12.9	15.8	24.0	20.6	24.1
para-Menth-1-ene-9-al	1217/1218	tr.	nd.	0.1	0.1	nd.	tr.	0.1	0.1	0.1	0.1
β-Cyclocitral	1219/1219	nd.	nd.	nd.	0.1	nd.	tr.	nd.	tr.	nd.	tr.
Nerol	1229/1229	0.1	nd.	0.1	0.1	nd.	0.1	0.1	0.1	0.1	0.1
Carvone	1243/1242	nd.	nd.	tr.	0.1	nd.	0.1	tr.	0.1	tr.	tr.
Geraniol	1252/1253	nd.	0.2	0.1	0.7	nd.	0.3	0.1	0.3	0.1	0.2
para-Anisaldehyde	1252/1254	0.8	nd.	0.5	nd.	nd.	0.1	1.1	0.2	1.3	0.3
(2E)-Decenal	1263/1263	nd.	nd.	nd.	0.2	nd.	0.1	nd.	0.1	nd.	0.1
Geranial	1268/1268	nd.	nd.	tr.	nd.	nd.	nd.	nd.	tr.	nd.	0.1
Ethyl salicylate	1269/1270	0.1	nd.	tr.	0.1	nd.	0.1	tr.	tr.	tr.	nd.
Nonanoic acid	1270/1271	nd.	nd.	nd.	nd.	nd.	0.2	nd.	0.5	nd.	0.4
(2E,4Z)-Decadienal	1292/1293	nd.	nd.	nd.	0.1	nd.	nd.	nd.	nd.	nd.	nd.

Tridecane	1300/1300	nd.	tr.	nd.	tr.	nd.	0.1	nd.	nd.	nd.	nd.
Undecanal	1302/1304	tr.	tr.	0.1	0.4	nd.	0.2	0.1	0.3	0.1	0.2
(2E,4E)-Decadienal	1316/1318	nd.	nd.	tr.	0.3	nd.	0.1	tr.	0.1	tr.	0.1
(3Z)-Hexenyl tiglate	1321/1320	nd.	nd.	nd.	0.1	nd.	tr.	tr.	tr.	tr.	tr.
Unknown		nd.	nd.	nd.	0.1	nd.	tr.	nd.	nd.	tr.	0.1
2-Undecenal	1376/1277	nd.	nd.	nd.	0.2	nd.	tr.	tr.	tr.	nd.	0.1
(E)-β-Damascenone	1383/1383	0.1	0.1	0.2	0.3	0.1	0.1	0.1	0.2	0.2	0.2
Unknown	-/1387	0.1	0.1	0.1	0.4	nd.	0.2	0.1	0.2	0.1	0.2
β-Elemene	1390/1390	nd.	nd.	nd.	0.1	nd.	tr.	nd.	tr.	nd.	tr.
Dodecanal	1408/1410	tr.	nd.	tr.	0.1	nd.	tr.	tr.	0.1	tr.	0.1
Unknown	-/1415	nd.	nd.	0.1	0.1	nd.	tr.	0.1	0.1	nd.	0.1
(E)-Caryophyllene	1419/1420	tr.	nd.	nd.	0.1	nd.	tr.	tr.	tr.	tr.	tr.
Neryl acetone	1420/1455	tr.	tr.	tr.	0.2	nd.	0.1	tr.	0.1	tr.	0.1
Dodecanol	1466/1467	nd.	nd.	tr.	0.1	nd.	tr.	tr.	tr.	tr.	0.1
(E)-β-Ionone	1487/1487	tr.	tr.	tr.	0.2	nd.	0.1	tr.	0.1	tr.	0.1
β-Selinene	1490/1490	nd.	nd.	nd.	0.1	nd.	nd.	nd.	nd.	nd.	nd.
Benzyl tiglate	1497/1498	tr.	nd.	tr.	tr.	nd.	tr.	tr.	0.1	tr.	0.1
(E,E)-α-Farnesene	1505/1505	nd.	tr.	nd.	0.2	nd.	tr.	nd.	nd.	nd.	tr.

Tridecanal	1510/1510	tr.	tr.	tr.	0.2	nd.	0.1	tr.	0.2	0.1	0.1
Unknown		nd.	0.1	0.1	0.5	nd.	0.2	0.1	0.2	0.1	0.2
(E)-Nerolidol	1563/1564	nd.	nd.	nd.	0.1	nd.	tr.	nd.	tr.	tr.	tr.
(3Z)-Hexenyl benzoate	1566/1567	nd.	nd.	nd.	0.1	nd.	tr.	nd.	tr.	tr.	0.1
Caryophyllene oxide	1583/1585	nd.	nd.	nd.	0.1	nd.	nd.	nd.	0.1	nd.	0.1
2-Phenyl ethyl tiglate	1585/1586	nd.	nd.	nd.	0.1	nd.	tr.	tr.	nd.	tr.	nd.
Tetradecanal	1612/1610	tr.	nd.	tr.	0.1	nd.	tr.	tr.	0.1	tr.	tr.
T-Muurolol	1642/1643	nd.	nd.	nd.	nd.	nd.	tr.	nd.	0.1	nd.	0.1
α-Cadinol	1650/1650	nd.	nd.	nd.	nd.	nd.	nd.	nd.	nd.	nd.	0.1
Tetradecanol	1675/1676	nd.	nd.	nd.	0.1	nd.	tr.	nd.	0.1	nd.	nd.
Heptadecane	1700/1700	nd.	nd.	tr.	0.1	nd.	tr.	tr.	0.1	tr.	0.1
Pentadecanal	1715/1714	nd.	nd.	tr.	0.1	nd.	tr.	tr.	0.1	tr.	0.1
Benzyl benzoate	1760/1760	tr.	nd.	0.1	0.2	nd.	tr.	tr.	tr.	tr.	tr.
Anthracene	1786/1786	nd.	nd.	nd.	0.1	nd.	tr.	nd.	nd.	tr.	nd.
Octadecane	1800/1800	nd.	nd.	nd.	0.1	nd.	tr.	nd.	tr.	nd.	nd.
Phytone	1841/1842	tr.	tr.	0.1	0.3	nd.	tr.	tr.	0.1	0.1	0.1
Benzyl salicylate	1864/1864	nd.	nd.	0.1	nd.	nd.	nd.	nd.	nd.	nd.	nd.
Hexadecanol	1883/1884	tr.	nd.	nd.	nd.	nd.	0.1	nd.	0.1	0.1	0.1

Nonadecane	1900/1900	tr.	nd.	nd.	0.1	nd.	tr.	nd.	nd.	nd.	0.1
2-Heptadecanone	1902/1902	0.5	0.2	0.9	1.3	0.7	0.3	0.9	1.3	1.3	1.4
Unknown		nd.	nd.	nd.	0.1	0.1	nd.	nd.	nd.	nd.	nd.
Unknown		nd.	nd.	nd.	nd.	0.1	nd.	nd.	nd.	nd.	nd.
Unknown		nd.	nd.	nd.	nd.	0.1	nd.	nd.	nd.	0.2	nd.
Eicosane	2000/2000	0.1	tr.	0.1	tr.	0.1	nd.	nd.	nd.	nd.	nd.
2-Nonadecanone	2090/2091	tr.	0.1	0.3	tr.	0.2	0.3	0.2	0.5	0.4	0.1
Unknown		nd.	nd.	nd.	0.7	0.1	nd.	0.1	nd.	nd.	0.3
Heneicosane	2100/2100	nd.	0.8	nd.	0.1	1.7	nd.	0.1	0.2	nd.	0.2
Phytol	2106/2106	tr.	nd.	0.1	0.2	nd.	0.1	0.1	0.2	0.1	0.2
Unknown		nd.	nd.	1.6	0.6	nd.	0.2	nd.	0.3	0.1	0.2
Docosane	2200/2200	nd.	nd.	0.8	8.4	0.1	0.8	tr.	2.5	0.8	2.2
Unknown		nd.	nd.	nd.	nd.	0.1	nd.	nd.	0.2	nd.	nd.
Tricosane	2300/2300	nd.	nd.	nd.	0.7	0.2	0.1	1.8	0.4	nd.	0.2
Tetracosane	2400/2400	nd.	nd.	0.7	5.9	1.0	0.3	0.7	0.9	nd.	0.7
**Total**		**98.8**	**99.3**	**98.8**	**96.6**	**100.0**	**98.8**	**98.9**	**98.4**	**99.0**	**99.0**
Monoterpene hydrocarbons		0.1	0.2	0.1	0.1	0.1	0.1	0.1	0.1	0.1	0.1
Oxygenated monoterpenes		0.7	0.7	1.1	2.5	0.6	0.5	0.9	2.3	1.2	1.5

Sesquiterpene hydrocarbons				0.5						
Oxygenated sesquiterpenes				0.2				0.2		0.3
Simple phenols	94.0	94.8	89.1	64.2	92.8	89.9	89.5	82	88.7	83.6
Aldehydes	2.4	2.2	2.7	7.0	1.9	4.6	3.0	4.8	4.2	5.0
Alcohols			0.1	0.8		0.4	0.1	0.6	0.2	0.7
Ketones	0.5	0.3	1.2	1.6	0.9	0.7	1.1	1.9	1.7	1.6

$RI^{Lit.}$: retention indices (Adams, 2007); $RI^{Calc.}$: retention indices relative to C_7-C_{30} *n*-alkanes on Rxi-5MS column; nd. – not detected.
